# Superior Sagittal Sinus: A Review of the History, Surgical Considerations, and Pathology

**DOI:** 10.7759/cureus.4597

**Published:** 2019-05-03

**Authors:** Tye Patchana, Bailey Zampella, James A Berry, Shokry Lawandy, Raed B Sweiss

**Affiliations:** 1 Neurosurgery, Riverside University Health System Medical Center, Moreno Valley, USA

**Keywords:** superior sagittal sinus anatomy, venous sinus thrombosis, dural sinuses

## Abstract

A systematic PubMed and Google Scholar search for studies related to the anatomy, history, surgical approaches, complications, and diseases of the superior sagittal sinus was performed. The purpose of this review is to elucidate some of the more recent advances of our understanding of this structure. One of the earliest anatomical landmarks to be described, the superior sagittal sinus (SSS, sinus sagittalis superior (Latin); “sagittalis” Latin for ‘arrow’ and “sinus” Latin for ‘recess, bend, or bay’) has been defined and redefined by the likes of Vesalius and Cushing. A review of the various methods of approaching pathology of the SSS is discussed, as well as the historical discovery of these methods. Disease states that were emphasized include invasion of the SSS by meningioma, as well as thrombosis and vascular malformations.

## Introduction and background

Historical perspective

One of the earliest anatomical landmarks to be described, the superior sagittal sinus (SSS, sinus sagittalis superior (Latin); “sagittalis” Latin for ‘arrow’ and “sinus” Latin for ‘recess, bend, or bay’) has been defined and redefined by the likes of Vesalius and Cushing [1]. Between the fall of the Roman Empire leading to the Dark Ages and the resurgence of intellectual inquiry, anatomical science underwent relatively little innovation. The Renaissance saw a resurgence of anatomical studies with the allowance of dissection for the purposes of scientific inquiry, including the injection of wax into the ventricles to map the anatomy of the brain [2]. Once anatomical structures could be defined, the study of pathology of those structures could commence. In 1888, Gowers was the first to describe dural venous sinus thrombosis, noting that “children are sometimes seized with hemiplegia, commencing with unilateral convulsions that are prone to recur” [3].

In 1932, Olivecrona classified the SSS into three divisions based on his studies of meningiomas [4]. In their 1938 monograph on meningiomas, Cushing and Eisenhardt first described the notion that resection at the anterior third of the SSS was a safe endeavor, during their discussion on the resection of parasagittal meningiomas. Cushing theorized that the resectability of this initial section of the SSS compared to the remaining two thirds may be due to the importance of venous drainage to the Rolandic Area [5]. Indeed, performing operations on the middle third of the SSS places both eloquent areas of brain as well as the bridging veins at risk [6]. That disruption of the anterior third of the SSS produces different postoperative morbidity is now a commonly held principle in neurosurgery. Even today, with knowledge that subtotal resection is associated with tumor recurrence, invasion of the SSS by tumors is considered by some to be a contraindication to complete resection. Others recommend complete resection with reconstruction of the venous structures [7]. Still others have in the past proposed allowing the SSS to become completely occluded so that collateral circulation may develop, allowing the sinus to be more safely resected [1]. Most recently, the SSS has been considered an operable anatomical structure, as long as meticulous surgical care is undertaken in its manipulation.

## Review

Anatomical and surgical considerations

The embryological formation of the cerebral venous system has been said to be a more complex process than that of the arterial system. In 1915, Streeter performed detailed serial sections of human embryos, some of which had undergone injection of the developing blood vessels with India ink and/or Berlin blue dye. His studies found that venous system precursors undergo formation between the 3rd and 4th weeks with formation of a capillary plexus, itself sprouting from the aortic arch. This plexus will go on to closely associate with the neural tube in a caudal to rostral fashion. Concomitantly, anterior, middle, and posterior dural plexus undergo formation that drain the neural tube into the head sinus, located laterally. The head sinus drains into the anterior cardinal vein, which will go on to contribute to the formation of the internal jugular veins. Between the 5th and 6th weeks post-gestation, eventual formation of anterior, middle, and posterior sinuses connected by a longitudinal sinus will drain into the internal jugular vein. Ultimately, Streeter concluded that the sagittal sinus will be formed by a section of the anterior venous plexus (also known as the plexus sagittalis) [8-9].

At 21 mm crown rump length (CRL), Streeter found that formation of a plexus sagittalis could be found as a subdivision of the anterior dural plexus. Earlier, at 14 mm CRL, large tributaries of anterior and middle dural plexus do not quite meet the median line. The exception to this is more anteriorly, where tributaries merge into a longitudinal plexus that is situated between the developing hemispheres. As the telencephalon grows posteriorly, the venous channels grow along with it and are stretched in the sagittal direction, leading to the orientation of the cortical draining veins in the adult. At this point, larger tributaries lead to formation of the primitive SSS in its simplest form. Streeter believed that it is not until later than 20 mm CRL that one may speak of a superior sagittal sinus proper - when the plexuses have fused at midline to form a longitudinal network. Additionally, he noted that the tendency for the SSS to drain on one side more than the other occurs at the earliest stages in the embryo and proceeds into adulthood [9].

Today, the origin of the SSS is typically described in posterior relation to the foramen caecum - at the intersection of the frontal bone and the ethmoid [10]. The SSS is often encountered during neurosurgical approaches for parasagittal and falx meningiomas, as well as interhemispheric transcallosal approaches, among many other access points into the skull. The need for safe surgical entry points into the cranium has led to a search for surface landmarks of the skull to orient surgeons to the architecture of the nervous system within. The SSS is no exception to this, and it has long been held that the sagittal suture of the skull may be used to approximate the location of the SSS. Studies involving cadavers have shown that the SSS typically lies to the right of the sagittal suture, albeit never more than 11 mm [10-11]. This has implications for placement of burr holes, as drilling within 1 cm of the sagittal suture has potential for disrupting the SSS. Knowledge of anatomical variations from traditionally taught neuroanatomy is important for neurosurgical planning.

It has been suggested that the SSS could be used as a surrogate measurement for transmural pressure, based on its size and has shown some correlation to what would be expected in some conditions [12-13]. This would be especially useful in the management of aneurysms. Deviation from normal surface landmarks is also useful in pathologies involving the skull. In young patients with unilateral coronal synostosis, anatomical deviation of the SSS has been found to occur. A study evaluating 50 children with unilateral coronal synostosis demonstrated that the SSS usually deviated contralaterally to the closed coronal suture and recommended a distance of 37 mm from the sagittal suture as a safe distance during surgical entry into the calvarium [14].

Exposure of the dural venous sinuses is often required in the management of lesions situated in proximity to the falx and tentorium. An array of surgical techniques exists for exposing the dural sinuses. Most of these techniques involve placement of burr holes lateral to the sinus but drilling directly over the sinuses has been described as well [15-16]. During removal of underlying bone, there is risk of injuring the sinuses (with possible complications of bleeding, air embolism, or venous infarct) due to the tight adherence of dura to the inner table of the skull. Gordon et al. identified five major techniques employed in craniotomies overlying the dural venous sinuses [12]. One of the newest techniques involves placement of anterior and posterior troughs positioned perpendicular to the sinus until dura is exposed laterally on each side. A footplate is then used to separate dura from the calvarium by drilling laterally from one trough, then back to connect with the other trough to produce a bone flap. This bone flap is then elevated from dura at the side farthest from the dural venous sinus, employing counterforce on the opposite side. The authors then recommend placing dural tack-up sutures around the perimeter of the craniotomy. This approach has been used on 82 cases spanning 2007-2015 without intraoperative complications or injury to the dural venous sinuses [17]. The SSS as a location for diverting cerebrospinal fluid (CSF) has also been evaluated. A recent case series involving lateral ventriculo-superior sagittal sinus shunting for hydrocephalus has been published, describing seven patients with hydrocephalus who had failed ventriculoperitoneal shunting, with good results [6].

Diseases Involving the Superior Sagittal Sinus

The superior sagittal sinus, owing to its location, is susceptible to a myriad of diseases, including invasion by meningiomas, thrombosis, arteriovenous fistulas, and more recently neurodegenerative proteinopathies [18]. The majority of research centers around causes, radiographic evaluation of, and treatment of SSS thrombosis or invasion by meningiomas. Additionally, secondary conditions or comorbidities related to pathologic involvement of the SSS are also important, including septic dural sinus thrombosis and an array of thrombophilias. Intracranial hypertension, pseudotumor cerebri, and sinus pericranii (abnormal connections between the intracranial and extracranial venous systems), are among associated conditions that may also affect this structure [19-22].

Meningioma Involvement in the Superior Sagittal Sinus

Much experience has been garnered from surgical considerations of removing meningiomas that have invaded the SSS. In the operative treatment of meningiomas invading the SSS, the advantages of a total resection must be balanced against the complications associated with surgery. Surgery for meningioma invasion into the SSS has been associated with postoperative venous sinus thrombosis leading to a conservative course of care with these tumors [23]. With preservation of the cortical veins (responsible for collateral drainage), complete resection of meningiomas invading the SSS has been advocated by some neurosurgeons [24]. Further, risks associated with aggressive surgery of the SSS can be avoided with sinus reconstruction [25].

A recent systematic review of aggressive vs non-aggressive resection of meningiomas of the SSS found that recurrence rates were not substantially different in the two groups. However, an aggressive tumor removal from the SSS was associated with higher venous bleeding and worsening of pre-existing motor deficits [26]. A variety of classification systems have been developed to describe the degree of invasion of the dural venous sinuses, including Sindou (which provides both grading and surgical treatment method), Merrem-Krause, and Bonnal- Brotchi classifications. The Sindou classification encompasses Type I, with meningioma attachment to the outer sinus wall, to Type VI with total occlusion of the sinus. An analysis of meningiomas by Sindou comes to the conclusion that whenever possible, total removal with sinus reconstruction or venous bypass is to be favored [27].

Superior Sagittal Sinus Thrombosis

With an incidence of 3-4 million per year, thrombosis of the SSS is associated with Virchow’s triad of hypercoagulability, venous stasis, and endothelial damage. Initial presenting symptoms similar to stroke in a young patient should raise suspicion for SSS thrombosis. Presentation can be variable, but usually includes focal neurological deficits (such as hemiparesis), signs and symptoms of intracranial hypertension, seizure, and encephalopathy [28]. Approximately one-third of cerebral venous thrombosis results in intracerebral hemorrhage [29]. The most frequently identified risk factors have been oral contraceptives in the presence of coagulation disorders, though 20-30% of cases remain unexplained. With an additional association with pregnancy, women are affected at a rate of 3:1 in comparison to men [30]. Because there is little anatomical variation of the SSS, absence of opacification allows for relative ease in the diagnosis of thrombosis. The venous phase of cerebral angiography is especially useful for visualizing the sinuses [31].

The mainstay for treatment of acute cerebral vein thrombosis continues to be anticoagulation with either unfractionated or low molecular weight heparin, regardless of the presence of bleeding intracranially. Indeed, venous thrombosis is an exception to the rule of holding anticoagulation during the presence of intracranial bleeding. In a meta-analysis of four randomized controlled trials (RCTs), Al Rawahi et al. concluded that low molecular weight heparin (LMWH) is a safe and effective treatment for the management of acute cerebral venous thrombosis. LMWH was found to have a higher risk to benefit ratio than unfractionated heparin [32].

Arteriovenous Fistulas of the Superior Sagittal Sinus

The SSS is a region susceptible to involvement by dural arteriovenous fistulas (DAVFs). Though rare, these lesions have been reported in numerous case studies. Complex venous structures in the region of the SSS make microsurgery a complicated endeavor. Although difficult to treat secondary to a large amount of multiple arterial feeders and venous drainage, as well as midline location, treatment is usually focused upon endovascular treatments and stereotactic radiosurgery [33]. A recent systematic review of the literature evaluating the current state of endovascular treatment for DAVFs in the SSS region concluded that endovascular treatment can completely resolve fistulas and reverse venous shunting [34].

Venous Air Embolism

Venous air embolism is a rare but fatal complication that may occur during neurosurgical procedures in which the venous system is exposed with the head elevated above the heart [35]. This is an especially feared complication of the sitting position for sub-occipital craniotomy, though some neurosurgeons feel this is an overstated complication. Standard measures in the treatment of venous air embolism include lowering the head below the heart, putting the patient in the left lateral recumbent position to trap air within the right atrium, ventilation with 100% oxygen, and aspiration of air from the right atrium with a central venous catheter. Hyperbaric oxygen therapy has been recommended for the treatment of arterial air embolism, but has recently been described in the treatment of cerebral venous air embolism [34].

## Conclusions

The SSS is an important venous structure that is implicated in many diseases affecting the intracranial cavity. Surgical approaches and treatment options must take into account this structure in a way that protects the integrity of this venous space. Here, we have reviewed the historical aspects, anatomical and surgical considerations, and diseases affecting the structure including meningioma, thrombosis, and vascular malformations.

Future research should continue to make this anatomical structure a more accessible and safe neurosurgical target. Case series discussing treatment of thrombosis, arteriovenous fistulas, and SSS co-morbidities may one day match those reported for meningioma involvement of the SSS.
